# Human Polyomavirus JCPyV and Its Role in Progressive Multifocal Leukoencephalopathy and Oncogenesis

**DOI:** 10.3389/fonc.2019.00711

**Published:** 2019-08-08

**Authors:** Luis Del Valle, Sergio Piña-Oviedo

**Affiliations:** ^1^Department of Pathology and Stanley S. Scott Cancer Center, Louisiana State University Health, New Orleans, LA, United States; ^2^Department of Pathology, College of Medicine, University of Arkansas for Medical Sciences, Little Rock, AR, United States

**Keywords:** JCPyV, progressive multifocal leukoencephalopathy, PML, brain tumors, Glioblastoma

## Abstract

The human neurotropic virus JCPyV, a member of the *Polyomaviridiae* family, is the opportunistic infectious agent of Progressive Multifocal Leukoencephalopathy (PML), a fatal disease seen in severe immunosuppressive conditions and, during the last decade, in patients undergoing immunotherapy. JCPyV is a ubiquitous pathogen with up to 85% of the adult population word-wide exhibiting antibodies against it. Early experiments demonstrated that direct inoculation of JCPyV into the brain of different species resulted in the development of brain tumors and other neuroectodermal-derived neoplasias. Later, several reports showed the detection of viral sequences in medulloblastomas and glial tumors, as well as expression of the viral protein T-Antigen. Few oncogenic viruses, however, have caused so much controversy regarding their role in the pathogenesis of brain tumors, but the discovery of new Polyomaviruses that cause Merkel cell carcinomas in humans and brain tumors in racoons, in addition to the role of JCPyV in colon cancer and multiple mechanistic studies have shed much needed light on the role of JCPyV in cancer. The pathways affected by the viral protein T-Antigen include cell cycle regulators, like p53 and pRb, and transcription factors that activate pro-proliferative genes, like c-Myc. In addition, infection with JCPyV causes chromosomal damage and T-Antigen inhibits homologous recombination, and activates anti-apoptotic proteins, such as Survivin. Here we review the different aspects of the biology and physiopathology of JCPyV.

## Introduction

The human neurotropic virus JCPyV is a small non-enveloped, double-stranded DNA virus that belongs to the family *Polyomaviridiae*. According to the latest taxonomic classification of the International Committee on Taxonomy of Viruses, there are 77 different species of Polyomaviruses that are divided in 4 genera, in addition to 3 others that could not be classified (1). They infect a wide variety of birds and mammals (2). However, of these 80 described Polyomaviruses, only 13 species have been identified to infect humans (3, 4), notably JCPyV, BKPyV, SV40, and Merkel Cell Polyomavirus. Two recently discovered Polyomaviruses will, most likely, be included in subsequent classifications (5, 6), bringing the number of Polyomaviruses affecting humans to 15. Table 1 shows these 15 human Polyomaviruses. JCPyV is of particular importance, as it is the known opportunistic infectious agent of the fatal demyelinating disease Progressive Multifocal Leukoencephalopathy (PML) (7).

**Table 1:** Human Polyomaviruses.

**Genus**	**Species**	**Name**	**Abbreviation**	**Discovery**	**Associated disease**
Alphapolyomavirus	Human Polyomavirus 5	Merkel Cell Polyomavirus	MCPyV	2008	Merkel Cell Carcinoma
Alphapolyomavirus	Human Polyomavirus 8	Trichodysplasia Spinulosa Polyomavirus	TSPyV	2010	Trichodysplasia Spinulosa
Alphapolyomavirus	Human Polyomavirus 9	Human Polyomavirus 9	HPyV9	2011	None known
Alphapolyomavirus	Human Polyomavirus 12	Human Polyomavirus 12	HPyV12	2013	None known
Alphapolyomavirus	Human Polyomavirus 13	New Jersey Polyomavirus	NJPyV	2014	None known
Betapolyomavirus	Human Polyomavirus 1	BK Polyomavirus	BKPyV	1971	Nephropathy/Hemorrhagic Cystitis
Betapolyomavirus	Human Polyomavirus 2	JC Polyomavirus	JCPyV	1971	Progressive Multifocal Leukoencephalopathy
Betapolyomavirus	Human Polyomavirus 3	KI Polyomavirus	KIPyV	2007	None/Acute Respiratory Symptoms
Betapolyomavirus	Human Polyomavirus 4	WU Polyomavirus	WUPyV	2007	None/Acute Respiratory Symptoms
Deltapolyomavirus	Human Polyomavirus 6	Human Polyomavirus 6	HPyV6	2010	Pruritic and Dyskeratotic Dermatosis
Deltapolyomavirus	Human Polyomavirus 7	Human Polyomavirus 7	HPyV7	2010	Epithelial Hyperplasia
Deltapolyomavirus	Human Polyomavirus 10	MW Polyomavirus	MWPyV	2012	None known
Deltapolyomavirus	Human Polyomavirus 11	STL Polyomavirus	STLPyV	2012	None known
–	Human Polyomavirus 14	Lyon IARC Polyomavirus	LIPyV	2017	None known
–	N/A	Mexican Polyomavirus	MXPyV	2012	None/Acute Diarrhea

While some new viruses, such as HIV-1 have been introduced to the human population very recently in our evolution (8), JCPyV has been a constant human companion for millions of years, most likely since prehistoric times (9, 10). Anthropologists use the genotype and strain of JCPyV carried by populations in order to trace ancient human migrations (11, 12). Based on their specific genotype, JCPyV strains have been classified in 8 types. Types 1 and 4, which are closely interrelated to each other have been found, not only in Europe, but in indigenous populations living in Japan, Siberia and Canada; Types 3 and 6 are characteristic of sub-Saharan Africa; types 2 and 7 have been isolated in with a large geographical distribution with subtype 2A present in Japan, 2B in Eurasians, 2D in India, and 2E in Australia; and subtypes 7A in Southeast Asia and 7B in Northern China and Mongolia, and finally, type 8 has been isolated in Papua, new Guinea and the Pacific Islands (9). A recent study that genotypes JCPyV DNA from semen samples showed an overall prevalence of 27.6% of viral DNA; however, up to 10.6% of these individuals had 2 different genotypes, suggesting that the first one was from their continent of origin and the second one was acquired in their destination country (13). Utilizing the study of their specific types and point mutations has shown close JCPyV proximities between native American and people from North-East Asia helping corroborate the migration of American ancestors across the Bering strait (14). It has also been demonstrated that JCPyV type 6 is the putative ancestral genotype, giving rise to two independent evolutionary lineages, one that includes types 1 and 4, and the other that includes types 2, 3, 7 and 8, which suggests not one, but two different migrations of *Homo sapiens* out of Africa, one to Europe, and the other to the Middle East and Asia (15). However, despite of our co-evolution with JCPyV since ancestral, pre-historic times, we were not aware of the presence of Polyomaviruses in humans until ~60 years ago, thanks to the first description of PML and the discovery of JCPyV.

### History of PML and the Discovery of JCPyV

The first characterization of Progressive Multifocal Leukoencephalopathy as an entity was done by Åström et al., who was the neuropathologist at Massachusetts General Hospital, when in 1958 they described an unusual demyelinating disease affecting multiple foci of the subcortical white matter, as a fatal complication in two patients suffering from chronic lymphocytic leukemia and one with Hodgkin's lymphoma (16). Within the plaques of demyelination, Richardson described the presence of perivascular cuffs of lymphocytes, enlarged oligodendrocytes with eosinophilic intranuclear inclusions, and bizarre astrocytes, which as he recorded, were so atypical, they reminded him of neoplastic cells. However, PML remained an extremely rare condition, and its etiology and pathogenesis a mystery. A year later, because of the inclusion bodies in oligodendrocytes, Cavanagh suggested for the first time a viral etiology hypothesis (17), that was reintroduced later on by Richardson himself (18). However, the first evidence of an infectious agent was presented at the symposium “Infections of the Nervous System” of the Association for Research in Nervous and Mental Diseases by Zu Rhein and Rubinstein who showed viral particles in the oligodendroglial viral inclusions by electron microscopy (19, 20), observation that was corroborated in every case, and the virus was classified in the family of *Papovaviridiae*, which at the time, because of their genomic similarities, included Polyomaviruses and Papillomaviruses. The Papova family has since disappeared and the present time Polyoma and Papillomaviruses are in their own separate families.

The next step in the consolidation of PML as an infectious disease did not come until 1971, as the virus proved challenging to cultivate *in vitro*. The high neurotropism of JCPyV was not known at the time, and viral replication failed in cultures of epithelial cells and fibroblasts. It was not until 1971 when Padgett, working in Zu Rhein's laboratory succeeded in replicating JCPyV in primary cell cultures of fetal glial cells (21), using extracts from the brain of a patient who had recently died of PML and had donated his brain. The initials of the patient's name were J.C., which is where the name of the virus originated. Of note, there has recently been an increasing numbers of manuscripts that include the name of this patient, instead of its official name (JCV or JCPyV), which, as Imperiale very eloquently put it, constitutes a violation of patient privacy and ethical principles and is a practice that should be stopped (22).

In order to fulfill the Koch postulates for infectious diseases (23), PML needed to be reproduced in an animal model, which brought the next serendipitous findings and the first evidence on the oncogenicity of JCPyV. During the last years of the 1970's and through the 80's JCPyV was inoculated into the brain of various animal models in an attempt to reproduce PML, however, to everyone's surprise, none of the different species of animals inoculated developed plaques of demyelination, and instead, a high percentage of them developed brain tumors. The species inoculated and the phenotype of tumors developed will be discussed later. Also in the mid 1980's, while the animal experiments were taking place, several detailed molecular studies resulted in the complete sequencing of JCPyV's genome (24).

### Structure of JCPyV

JCPyV is a small, double stranded, supercoiled, DNA virus, without an envelope, and covered by an icosahedral capsid. The prototype strain of JCPyV, designated Mad-1 (because it was first isolated in Madison, WI), measures ~40 nm in diameter and its genome is comprised of 5,130 nucleotides, that can be functionally divided into three regions (1, 24) a control, non-coding, regulatory region that separates; (2) an Early transcriptional region; and (3) a Late transcriptional region. The name of these two refers to the time of transcription during the viral cycle, before or after viral replication, respectively. The control region (CR), located in between the early and later regions, is comprised of a duplicated 98 base pair promoter, which allows for bidirectional reading and activation, counter-clock wise for early genes, and clockwise for the later genes (25). It also contains the sites for origin of transcription and binding to different transcription factors (NF-κB, NF-1, Sp1, EGR-1, YB-1, and Pur-α among others) (26, 27), that can activate JCPyV transcription. The CR is also important because of its genetic variability, which allows for the existence of many strains, each conferring JCPyV with different functional characteristics, species and cellular tropism, as well and pathogenicity (28, 29). For example, when comparing the pathogenic characteristic of the archetype strain, isolated from kidney, the wide variety of rearranged strains (30), isolated from PML brains show novel Spi-B binding sites, that upon activation, confer JCPyV with increased early gene expression and higher replication rates, resulting in increased cytopathology (31, 32). It has been postulated that such rearrangements in the CR occur in B-lymphocytes in the bone marrow, during genetic recombination (33).

The early transcriptional region, named so because its transcription occurs before viral replication, encodes for the regulatory proteins large T-Antigen (T-Ag), denominated “T” for its “tumoral” activity, and small t-Antigen (t-Ag). After the activation of the promoter, large T-Antigen is synthesized in cytoplasmic ribosomes, and quickly moves into the nucleus, where it binds to the promoter once again, stimulating the activation of the capsid proteins, VP1, VP2, and VP3. Large T-Antigen is a 708 aminoacid, 80 K-Dalton versatile protein that possess multiple functions, including Helicase, ATPase, and Phosphatase; it can keep JCPyV infected cells in S phase by binding and sequestering p53 and pRb (34, 35). All these functions are designed to efficiently orchestrate viral replication; however, they can also confer JCPyV with oncogenic properties. From the original large T-Antigen gene some variants of the protein can also be generated via splicing; these include small t-Antigen, T'_135_, T'_136_, and T'_165_, proteins whose functions are not completely known. However, it has been shown that small t-Antigen remains in the cytoplasm and improves the efficiency of viral replication and that it is important for cellular transformation (36). Also, the spliced proteins T'_135_, T'_136_, and T'_165_ have been shown to enhance T-Antigen induced viral replication in human cells (37), and to inactivate cell cycle regulators, inducing viral transformation and immortalization of cells (38).

The late transcriptional region encodes for the capsid proteins. Of these, VP1 is the largest and most abundant with 360 copies, organized in 72 pentamers and accounting for 80% of the capsid, while VP2 and VP3 are mostly binding proteins that constitute the remaining 20% (39). VP1 molecules, usually composed of 350 amino acids, contains a binding site that interacts with sialic acids attached to glycans, especially gangliosides, initiating the attachment of the virus and the infection of cells (40). On the other hand, VP2 and VP3 are unstructured proteins that keep the VP1 pentamers cohesive and have DNA-binding domains, which helps JCPyV entry into new host cells (41). Rearrangements in the capsid genes are also important in the pathogenesis of JCPyV. For example, mutations affecting the surface loops of VP1 result in escape of antibody-mediated neutralization and are a key factor in JCPyV evasion of the immune system (42). In addition, two novel Open Reading Frames (ORF1 and ORF2) were recently discovered in JCPyV strains isolated from PML patients. These ORFs are generated by an unusual splicing process called trans-splicing, that occurs on the 5'-short coding regions of VP1, between the genes for the Agnogene and the VP2 gene. These frames encode for 2 proteins of 58 and 72 amino-acids, respectively, and blocking of transcription of these proteins results in significantly less efficient viral replication, providing a novel and potential target for future therapies (43).

**Figure 1:**
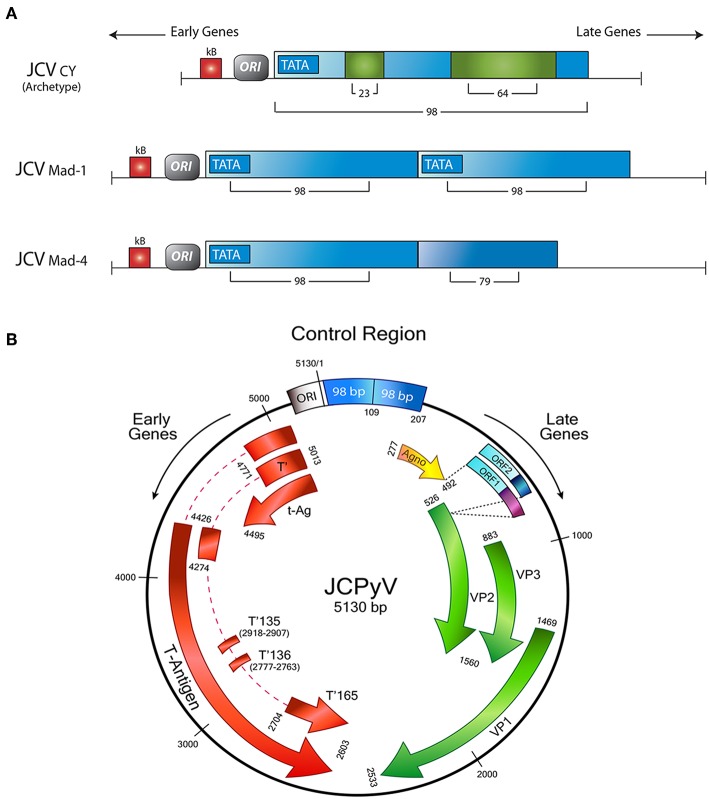
Schematic representation of the JCPyV genome. **(A)** The control regulatory region determines the strain of JCPyV. The archetype strain in composed of 98 base pairs, with 2 insertions of 23 and 64 respectively. The Mad strains are characterized for a duplication of the control region; Mad-1 contains two 98 base pairs, while in Mad-4, the second repeat has a deletion of 19 base pairs. **(B)** The control region divides the Early and Late genes. The Early transcriptional region encodes for Large T-Antigen, and the smaller splice versions, small t-Antigen, T'135, T'136, and T'165. On the other side, the late transcriptional region contains the genes for the accessory agnoprotein and the capsid proteins VP-1, VP-2, and VP-3. In addition two new Open Reading Frames have been discovered between the Agno y VP1 genes. Modified from original with permission from Del Valle et al. (48).

The late genes also encode for a small accessory product, of 71 amino acids, denominated Agnoprotein. The name of the protein derives from the Greek “agnosis,” which is translated as “without knowledge”; however, although earlier the function of Agnoprotein was not fully known, many recent studies demonstrated its importance for viral transcription and viral replication, as well as the transport of newly assembled virions from the nucleus to the cytoplasm (44, 45). It also acts as a viroporin, facilitating viral release (46). As it is the case with the CR, mutations in this area of the viral genome have serious implications for the biology of JCPyV. It has been shown that a strain with a deletion of 143 base pairs in the Agnogene, isolated from a patient with a newly described clinical condition called JCPyV granulopathy, result in the inability of JCPyV in establishing persistent infection (47). Figure 1 shows a schematic representation of the JCPyV genome, depicting all the regions and genes involved in the production of the proteins discussed, as well as variations in the control region, responsible for the viral strain.

### Epidemiology and Biology of JCPyV

The primary infection with JCPyV in humans in asymptomatic, which makes the age of primary infection difficult to ascertain, However, sero-epidemiological studies have shown that only 8–10% of children of 1–5 years old exhibit antibodies against JCPyV; however that number dramatically increases to 28% by age 10 and to 65% by age 14, strongly suggesting that the primary infection occurs in early childhood (49, 50). By adulthood the percentage of people that exhibits antibodies ranges from 80 to 95%, making JCPyV a widespread ubiquitous virus (51, 52). Traditional early sero-epidemiological studies relied on hemagglutination, which was later replaced by enzyme-linked immuno-absorbent assays; however, the close homology among viral proteins could lead to false positive results; a recent study, which uses a novel indirect ELISA methodology with synthetic peptides that mimic the JCPyV capsid protein VP-1 specifically, and thus, prevent cross-reactivity with other Polyomaviruses has corroborated the high rates of infection in healthy individuals (53). Another very interesting study has shown the presence of JCPyV genome in multipotent mesenchymal cells of the Wharton's jelly of the umbilical cord may suggest an even earlier age of exposure to the virus (54). Another mystery was the via of transmission; however, a series of breakthrough studies have shown the presence of JCPyV particles in raw urban sewage world-wide (55). This, in addition to the finding of viral genomic sequences in the upper and lower digestive tract strongly suggest a fecal-oral mode of transmission (56, 57). However, viral sequences are also found in lymphocytes of the Waldeyer's ring and the upper respiratory tract, which makes the respiratory route of infection also likely and not mutually excluding with the oral route (58, 59). Later on, JVPyV undergoes hematogenous dissemination and establishes latency in the bone marrow and kidney (60, 61). Slight immunodepressive states result in viral reactivation; for example, in pregnancy, or advanced age viral particles can be found shedding in the urine (62, 63). Different phenotypes of JCPyV have also been found in the urine of patients suffering from autoimmune diseases such as lupus erythematosus, Sjogren's syndrome, rheumatoid arthritis, and dermatomyositis (64–66). Of note, the strain of JCPyV that is responsible for the primary infection and establishes latency in normal individuals is the Archetype strain (CY), which contains only a single 98 base pair control region.

Once latency has been established, JCPyV can reach the brain carried by B-lymphocytes (67, 68) to either establish latency there or to cause PML; however the detection of JCPyV genomic sequences in autopsy brains from immunocompetent patients, may suggest yet another site for viral latency (69, 70). JCPyV then enters glial cells through sialic acid receptors and serotonin receptor 2A (5-HT_2A_-R) (71, 72). It was recently demonstrated that these interactions occur in a narrow window of time and that an intracellular loop of the receptor is necessary for infection and viral internalization by β-arrestin (73).

Interestingly, sialic acid receptors are abundant in kidney and colonic epithelial cells, lymphocytes and glial cells, which explains the range of tissues where JCPyV is commonly found (74). JCPyV can also enter endothelial cells, independently of these serotonin receptors, indicating yet another site for latency in the brain (75). Once the capsid protein VP-1 has established contact with the mentioned receptors, the virus enters the cell via clathrin-dependent endocytosis (76) and is transported into the nucleus by endosomes and caveosomes, through intermediate filaments and microtubules (77). Then, nuclear localization signals, also present in VP1 interact with importins, allowing JCPyV to enter the nucleus through the nuclear pore complex (78), where the capsid is disassembled and transcription and viral replication can take place in oligodendrocytes and astrocytes. The marker tropism of JCPyV for glial cells can be explained by the presence of glial-specific transcription factors that can bind to the CR, as described above (79). For example, the range of expression of Nuclear Factor 1 (NF-1) proteins class D, preferentially expressed in neural crest-derived cells, could be directly related to the tropism and activation of JCPyV, which has NF-1 binding sites (80, 81). Astrocytes undergo an abortive infection and remain, acquiring pleomorphism and a transformed phenotype with pleomorphism and nuclear atypia, while in oligodendrocytes, JCPyV infection, and the accumulation of viral particles in the nucleus, results in their lytic destruction and in the development of PML. It has been shown by quantitative genomic, proteomic, and molecular localization analyses, that once released, mature viruses can spread via myelin sheaths of the subcortical white matter, but as the demyelinated lesions reach the cortex, the spread does not continue in gray matter myelin sheaths (82).

Because of the high affinity of VP proteins with sialic acid receptors, and the high efficiency of viral entry into epithelial cells of the gastrointestinal and urinary tracts, and lymphocytes, as discussed above, JCPyV-like particles without viral genomic material, which are easy to engineer are now being used as delivery vectors for novel therapies. This novel way to use JCPyV as a tool started when these “capsoids” were successfully internalized *in vitro* in mouse fibroblasts and the delivered methotrexate intracellularly to leukemia T-cells (83). Later, these particles were used to deliver suicide genes intracellularly to mouse colon adenocarcinomas (84), diffuse large B-cell lymphomas (85), and a xenograft model of bladder cancer (86), successfully inhibiting tumor growth in all scenarios.

### Progressive Multifocal Leukoencephalopathy

Before the AIDS pandemic in the early 80's, there were only 238 cases of PML reported in the literature, the majority in patients with leukemias, lymphomas and auto-immune diseases. However, the incidence of PML increased up to 20 times in the beginning of the 1990's (87). It was estimated that PML constituted ~5% of the neurological complications of patients with AIDS and that up to 85% of patients with PML are seropositive for HIV-1 (88, 89). The efficacy of highly active antiretroviral therapy (HAART) has resulted in a significant decrease in these numbers during the last two decades; however, a new peak of incidence has appeared due to the use of immune therapies, such as Rituximab, an anti-CD20 for the treatment of non-Hodgkin lymphoma, lupus and rheumatoid arthritis (90), and most notably, Natalizumab, an anti-α_4_β_1_ and α_4_β_7_ integrin for the treatment of Multiple Sclerosis and Crohn's disease (91, 92). However, the risk of developing PML under these treatments is still considered low, of ~1:1,000 patients in an average period of 18 months (93, 94). The neurological symptomatology of PML is related to the location and size of the demyelinating lesions. Frontal and parietal lobes are the most commonly affected, however lesions can occur in any supratentorial location, including the brainstem and cerebellum (95), and even the spinal cord (96, 97).

The signs and symptoms are diverse and complex, which makes the diagnosis of PML challenging, and include subcortical dementia, cognitive impairments, dysarthria, motor and sensory aphasias, ataxia, paresis, bradykinesia and in rare occasions paresthesias and seizures (98). No differences in symptomatology between AIDS-associated and non-associated PML have been established. Because of the variability in symptoms, a suspected diagnosis of PML has to be confirmed by Polymerase Chain Reaction (PCR) amplification of viral DNA by PCR on cerebrospinal fluid. With an 81–94% sensitivity and a 95–100% specificity, PCR amplification was the golden standard for the diagnosis, however the sensitivity has significantly decreased after the introduction of HAART therapy, due to the low copy number, consequence of the reconstitution of the immune system (99). But new PCR modalities, such a real time PCR have restored the sensitivity of the assay, with the advantage that the results seem to correlate with the prognosis (100, 101). On the other hand, the sensitivity and specificity in serum, peripheral mononuclear cells or urine is low and does not correlate with the disease (102). The prognosis of PML is somber with a life expectancy of ~6 months after diagnosis, however, a few cases of long survival and even remission have been reported in up to 10% of patients (103, 104). Interestingly, in the surviving group, CD8+ cytotoxic lymphocytes, specific against viral antigens have been demonstrated, and these are absent in fatal cases of PML (105, 106). An epitope of 9 amino-acids in the capsid protein VP-1 has been identified as target for such lymphocytes, which could have future potential prognostic applications (107).

**Figure 2:**
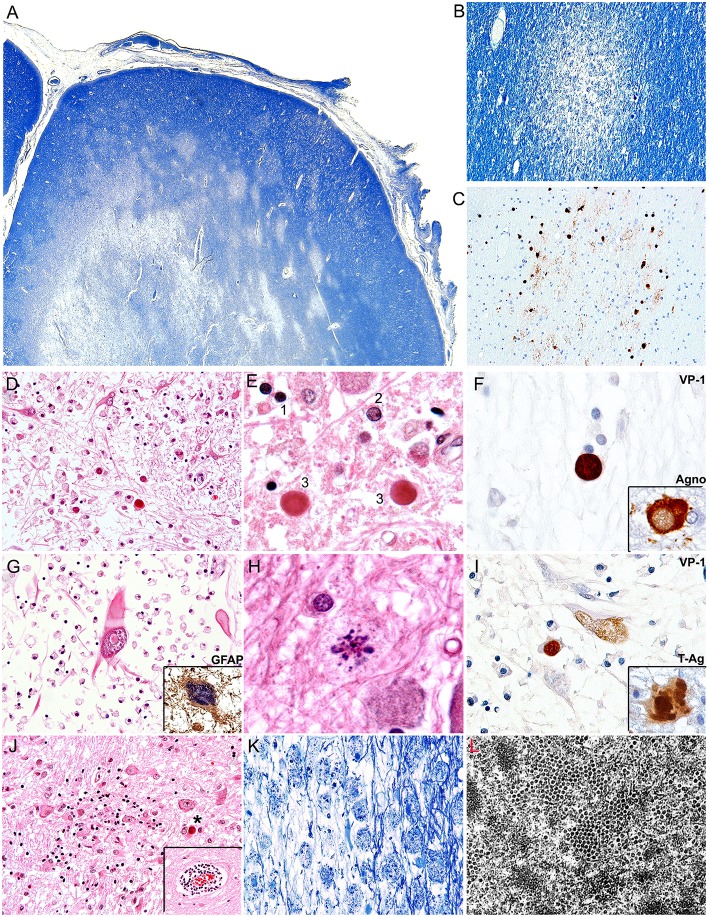
Histopathological features of PML. **(A)** Montage of a frontal lobe gyrus in a case of PML demonstrates extensive plaques of demyelination in the subcortical white matter (Luxol Fast Blue, 40x magnification). **(B)** At higher magnification, an early plaque shows well defined borders (Luxol Fast Blue, 100x magnification). **(C)** A consecutive section shows the same plaque or demyelination immunolabeled with a VP1 antibody, which highlights JCPyV infected oligodendrocytes in the periphery of the plaque (Original magnification 100x). **(D)** A plaque of demyelination showing numerous enlarged oligodendrocytes in the middle and lower field, and several bizarre astrocytes in the upper field (H&E, 400x magnification). **(E)** At higher magnification the different types of inclusion bodies are shown; an early inclusion (2), with 2 late, intensely eosinophilic inclusions (3) in comparison with a non-infected oligodendrocyte (1) (H&E 1,000x magnification). **(F)** Immunohistochemistry for viral proteins shows VP1 in the intranuclear inclusion bodies of oligodendrocytes and the accessory Agnoprotein in the cytoplasm. **(G)** A bizarre astrocyte with elongated cytoplasmic projections and an intranuclear inclusion body is shown. (H&E 600x magnification). Immunohistochemistry for GFAP corroborates their phenotype (insert, 600x magnification). **(H)** Atypical mitotic figures can also be found (H&E 1,000x magnification). **(I)** Immunohistochemistry for viral proteins demonstrates the nuclear expression of VP1 and the nuclear and cytoplasmic expression of the early protein T-Antigen. **(J)** Additional histological features include microglial nodules and perivascular cuffs of lymphocytes (insert) (H&E 200x magnification), and **(K)** foamy macrophages that phagocyte cellular debris and myelin released by the lysed oligodendrocytes (Luxol Fast Blue, 200x magnification). **(L)** An electro micrography shows abundant icosahedral viral particles in the intranuclear inclusion bodies of oligodendrocytes.

PML is radiologically characterized in CT scans by hypodense areas in the subcortical white matter, without enhancement. These lesions are asymmetrical, well-defined and tend to become confluent as the disease progresses. By MRI, the lesions are hypointense in T1 and hyperintense In T2 (108, 109). The main differential diagnosis is with multiple sclerosis, which tends to be deeper and in the vertices of the lateral ventricles, and in PML, the subcortical lesions tend to affect arcuate fibers (U fibers), creating a sharp border with the cortical gray matter. From the gross pathology point of view, PML is characterized by multiple plaques of softening, brown-gray to yellow-gray in color, of irregular, well-defined borders, which can be individual in early stages, confluent in later stages and even form cavitations (7). Histologically PML is characterized by three pathognomonic features, the mentioned subcortical demyelinated plaque, which can be highlighted by a special stain for myelin (Luxol Fast Blue), and within the plaques, the presence of enlarged oligodendrocytes harboring a large intranuclear eosinophilic inclusion bodies, which represent active viral replication and bizarre astrocytes. Depending on the number and maturity of the virions, the inclusion bodies can show as clear or slightly basophilic bodies that displace normal chromatin to the periphery of the nucleus (“early” inclusions), or large eosinophilic homogeneous inclusions that completely replace chromatin (“late” inclusions). Characteristically these infected oligodendrocytes are present in the borders of the demyelinated plaques. The final fate of the oligodendrocytes is to undergo lytic destruction. The atypical astrocytes demonstrate severe pleomorphism and they can contain one or several irregular nuclei with condensed chromatin and a prominent nucleolus, and they too can contain small inclusion bodies, both in the nucleus and the cytoplasm. The astrocytes do no undergo lysis. Additional features include numerous “foamy” macrophages, whose function is to phagocyte the myelin released by lysed oligodendrocytes and perivascular cuff of lymphocytes (7). Although these features are characteristic of PML, the diagnosis can be further confirmed by immunohistochemistry for viral proteins. Infected cells express the capsid protein VP-1, preferentially in the nuclei, but it can also be found in the cytoplasm. The early protein, T-Antigen is preferentially seen in the nuclei of astrocytes and oligodendrocytes, while the accessory product Agnoprotein, shows a cytoplasmic, perinuclear location in oligodendrocytes (110). By electron microscopy icosahedral viral particles can be seen in the nuclei of infected cells, where the virions assemble, however, these can also be associated to the endoplasmic reticulum, and the membrane, and they can assemble in a filamentous pattern (111, 112). Figure 2 depicts the common histological and immunohistochemical features of PML.

The development of PML depends on many factors, including the immune status of the individual, the co-infection with HIV-1, the viral strain, and the ability of JCPyV to reach the brain. It has been postulated that JCPyV is carried into the brain by lymphocytes, were it establishes latency (68, 113). Because PML occurs in such high prevalence in patients with AIDS, a cross-talk among both viruses has been investigated. It was then demonstrated that the HIV-1 transactivator protein Tat, can be secreted by infected macrophages that are not infected by JCPyV, and then internalized by JCPyV infected oligodendrocytes, binding to the control regulatory region, and activating its transcription and replication (114, 115). This suggests that co-infection by both viruses is not necessary for their interaction. In addition, an indirect mechanism of cross talk involving the Tat activation of cytokines, including TGF-β; when TGF-β binds to it receptor in the surface of JCPyV infected oligodendrocytes, it stimulates the activity of Smad 3 and Smad 4, which in turn bind to the promoter region of the virus, activating its transcription (116). These direct and indirect mechanisms are not mutually excluding. It has also been shown that activation of the JCPyV promoter can be increased by Tat itself (117) or by the hypoxia inducible factor alpha (HIF-1α) (118). Another mechanism on the physiopathology of PML may also be important in the oncogenesis of JCPyV, and involves the inhibition of apoptosis. While abundant reports have shown apoptotic death in a wide variety of encephalitis, there are no credible reports of apoptosis in PML at morphological or structural levels (119). On the contrary, it has been experimentally demonstrated that JCPyV induces non-apoptotic cell death in infected glial cells (120), and that JCPyV T-Antigen can activate the normally dormant promoter of the anti-apoptotic protein Survivin, resulting in the protection of infected cells from apoptosis, a mechanism normally used by eukaryotic organisms to dispose of damaged or virally infected cells (121).

While there is no effective treatment for this fatal disease, 2 new lines of studies have recently offered hope in limiting PML. The first study, which was done *in vitro*, involved gene editing using the CRISPR/Cas 9 system to target the JCPyV genome areas that serve as sites for the creation of guide RNAs for T-Antigen. This clever approach, which has proven effective in other viral infections, resulted in the suppression of viral replication in infected permissive cells and pointed to gene editing as a potential novel tool in the treatment of PML (122). The second line of research was done *in vivo* and involves targeting PD-1, a negative regulator of the immune system's response that contributes to impaired viral clearance; treatment of 8 PML patients with Pembrolizumab (123), an inhibitor of PD-1, resulted in the activation of CD4+ and CD8+ activity, reduction of JCPyV viral load and clinical improvement or stabilization of the disease in five of them, but no meaningful change was observed in the remaining three. These mixed results, however, point to immune checkpoint inhibitors as another potential treating agents for PML.

### Animal Studies

As described in the section regarding the history of PML, early studies performed in order to reproduce the disease in animal models resulted in the surprising development of brain tumors and not in plaques of demyelination. Direct inoculation of JCPyV in the brain of adult Syrian hamsters resulted in the development of medulloblastomas and pineocytomas in ~83% of animals (124, 125). Inoculation at birth also resulted in the induction of medulloblastomas at 3–6 months of age (126). Another study in which hamsters were inoculated intraocularly at birth described the presence of neuroblastomas in the mediastinum, pelvis, abdominal cavity and neck of 33% of animals (127). A change of animal model yielded similar results, with 20 of 27 rats developing primitive neuroectodermal tumors (PNETs) in the cerebrum after intracranial inoculation with the Tokio-1 strain of JCPyV (128, 129). The use of an animal model more closely related to humans offered no different outcome, and the inoculation of JCPyV intracerebrally, subcutaneously or intravenously in squirrel monkeys resulted in more than half of them developing glial tumors, ranging from astrocytomas to Glioblastomas 14–30 months after inoculation (130, 131). These studies showed T-Antigen expression by the tumor cells, but no production of capsids or viral particles, indicating that monkey cells are not permissive for JCPyV replication. Another non-human primate model also yielded similar results, with owl monkeys developing astrocytomas 16–36 months after direct inoculation of JCPyV into the brain parenchyma. This study was of particular importance because it demonstrated for the first time that viral integration into the cellular DNA had occurred in all tumors and in derived cell lines. In addition, the number of sites of integration was limited, suggesting a clonal origin for the tumors (132). All these models, although unsuccessful in replicating PML, provided the first and some of the strongest evidence into the oncogenicity of JCPyV.

**Figure 3:**
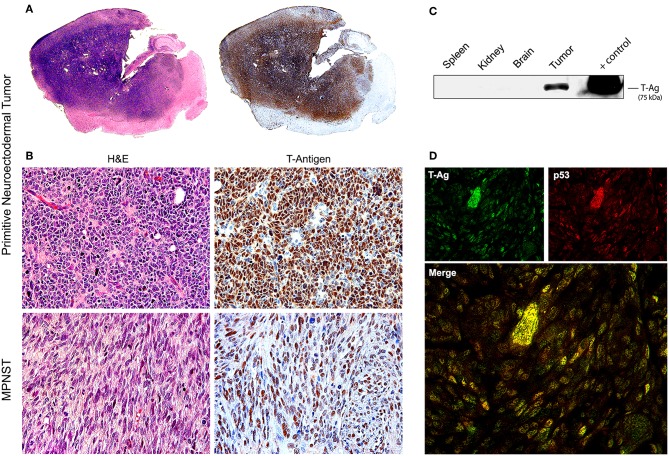
Tumors in transgenic mice. **(A)** Consecutive montages of the brain of a JCPyV transgenic mouse demonstrating a large blue, small-cell tumor, that practically substitutes the entire parenchyma. By immunohistochemistry, the entire tumor expresses T-Antigen, indicating the activity of the T-Antigen transgene. **(B)** At higher magnification, the tumor is composed of sheets of poorly differentiated cells with a marked lack of cytoplasm, indicating their embryonal phenotype. Immunohistochemistry for T-Antigen shows nuclear expression in the majority of neoplastic cells (Original magnification 400x). Histological pictures of a malignant peripheral nerve sheath tumor (MPNST) and its expression of T-Antigen are also shown. **(C)** A Western blot of different tissues of the mouse shows the expression of T-Antigen exclusively in the tumors. Although all cells from all tissue carry the transgene. **(D)** Double labeling immunofluorescence in a PNET shows co-localization of T-Antigen and wild-type p53 in the nuclei of neoplastic cells. Both proteins are expressed at different degrees in the tumor cells (Original magnification 600x).

Later, once such oncogenicity was attributed to the early protein, T-Antigen, and its ability to sequester key cell cycle proteins, more sophisticated models were created, specifically transgenic mice containing exclusively the early transcriptional region of JCPyV, that is, the T-Antigen coding region. These mice also spontaneously developed adrenal neuroblastomas (133). Another transgenic line, containing the early transcriptional region, under the regulation of its own promoter resulted in ~75% of mice developing massive primitive neuroectodermal tumors in the mesentery (134); once the colony expanded, other phenotypes of tumors derived from the neural crest were observed, including PNETs affecting the brain parenchyma (135), pituitary tumors (136), and in a small subset of animals, malignant peripheral nerve sheath tumors (MPNSTs) affecting the sciatic nerve and extremities (137). In these models it was demonstrated that, although the early genome is present in every cell of every tissue, T-Antigen was exclusively expressed by neoplastic cells, and not even in the adjacent normal brain, indicating the specificity of T-Antigen in the oncogenic process. It was also shown that T-Antigen and p53 are not only co-localized in neoplastic cells, but actually binding to each other. The close parallels between tumors developed by these transgenic models and human tumors, as we will discuss below, makes them excellent tools for the study of JCPyV oncogenesis. Figure 3 depicts examples of PNETs and MPNSTs in these transgenic animals, with prominent expression of T-Antigen in the nuclei of neoplastic cells.

### JCPyV in Human Brain Tumors

Even before the discovery of JCPyV, several reports demonstrated concomitant tumors in cases of PML. The first of them was reported by Richardson himself, in his second paper characterizing PML. This case was of a 58 year-old man suffering from chronic lymphocytic leukemia who developed PML; the post-mortem examination revealed an oligodendroglioma in addition to the demyelination (18). A few years later, two publications reported multiple astrocytic tumors in combination with PML. The first one with tumors in the cortex (138), and the second one of a patient who developed spontaneous PML, without evidence of immunosuppression, and presented multiple astrocytomas in the pons and cerebellar hemispheres (139). However, the first association of JCPyV with human brain tumors was reported in a 21 year-old male with common variable immunodeficiency, who died of PML after a 9 month clinical course. In addition to PML, the post-mortem examination revealed numerous foci of “dysplastic ganglion cells” in the temporal and occipital lobes, which showed expression of T-Antigen by immunohistochemistry and *in situ* hybridization for mRNA (140), implicating JCPyV in the transformation of these cells. This case, and the studies in transgenic mice, who's most commonly develop tumors are PNETs, open the door for studies in other human tumors. JCPyV was detected by PCR with primers for the carboxy- and amino-terminals of T-Antigen, as well as the VP1 region in 84% of the 38 cases studied. Most importantly, immunohistochemistry demonstrated the nuclear expression of T-Antigen in 57% of these tumors, all of them co-localizing with wild type p53 (141–143). Later on, the expression of the late product, Agnoprotein was also detected in these tumors (144). In one case, which was originally misdiagnosed as a supratentorial PNET, the expression of JCPyV T-Antigen was particularly widespread and robust. This was in reality the recurrence of a previously surgically excised Glioblastoma with a small cell component (48). A further case of this entity revealed the presence of JCPyV viral sequences and the expression of both, T-Antigen and Agnoprotein in both phenotypes of tumor cells, corroborating the hypothesis that these clonal tumors originate in a single precursor cell which then takes two pathways of differentiation (145). This study was also important because it was the first in which laser capture microdissection was used to amplify viral sequences from individual cells from both phenotypes.

These experiments, performed in single cases, opened the door for studies at a larger scale in human glial tumors. In one of the largest to date, 85 glial brain tumors were examined by PCR, utilizing three sets of primers, for the early, late and control regions, and by immunohistochemsirty. These tumors included oligodendrogliomas, astrocytomas, pilocytic astrocytomas, oligoastrocytomas, anaplastic astrocytomas, anaplastic oligodendrogliomas, glioblastomas, ependymomas, sub-ependymomas and 1 case of gliomatosis cerebri and 1 case of gliosarcoma. Viral sequences were detected in 49 of these samples (69%). The numbers for specific groups were as follows 83.3% in ependymomas, 76% in diffuse fibrillary astrocytomas, 80% in anaplastic gliomas, and 57.7% in glioblastomas. T-Antigen expression was found in 38.9% of the tumors, and for specific groups, 66.6% in ependymomas, 43.7% in diffuse fibrillary astrocytomas, 40% in anaplastic gliomas, and only 23% in glioblastomas. Interestingly, in the case of gliosarcoma, viral sequences and expression of T-Antigen were only detected in the glial phenotype of the tumor. Sequencing of the control region demonstrated that the strains of JCPyV present in these tumors are the Mad-1 and Mad-4 mainly (146). In another study performed in a collection of oligodendrogliomas from Mexico and the United States, similar results were shown by PCR and immunohistochemistry; however, sequencing of the control region revealed that the tumors from the United States contained genomic fragments of the Mad-4 strain, while the tumors from Mexico contained fragments from the archetype strain (147). These observations corroborate the anthropology findings into the geographical distribution of JCPyV and make the possibility of laboratory contamination very unlikely. Similar results were found in another study of glial brain tumors from Italy, and in that case Mad-4 was the predominant strain amplified (148). Interestingly, expression of the late capsid protein VP1- has not been detected in any of the studies, ruling out productive infection, and suggesting that the JCPyV may be integrated in the neoplastic cell DNA.

Although all these studies are carefully done and show clear results, the role of JCPyV in the pathogenesis of brain tumors has been debated. For example, it has been suggested that the detection of viral sequences by PCR may come from the tumor microenvironment, since JCPyV infects and is carried into the brain by lymphocytes, as discussed above; however, this was clarified by several studies in which PCR amplification with JCPyV probes was successfully performed in single tumor cells extracted from the tissue by laser capture microdissection (145, 149, 150). Other main concerns have been the possible contamination of samples and that the presence of viral DNA does not mean activity; however, the expression of T-Antigen, detected by immunohistochemistry in paraffin-embedded samples makes these arguments difficult to sustain. In an immunohistochemical study performed in commercially available tissue microarrays, T-Antigen protein expression was present in the nuclei of ~40% of the tumors samples (151). In recent year, however, the refinement of classic molecular biology techniques as well as the development of new, more sensitive and specific assays has resulted in a new wave of manuscripts detecting JCPyV in brain tumors (152–154). Among these better and more sensitive assays, a new cocktail of anti-T-Antigen antibodies from different Polyomaviruses was recently developed and can be used for specific detectionin tissue microarrays (155).

**Figure 4:**
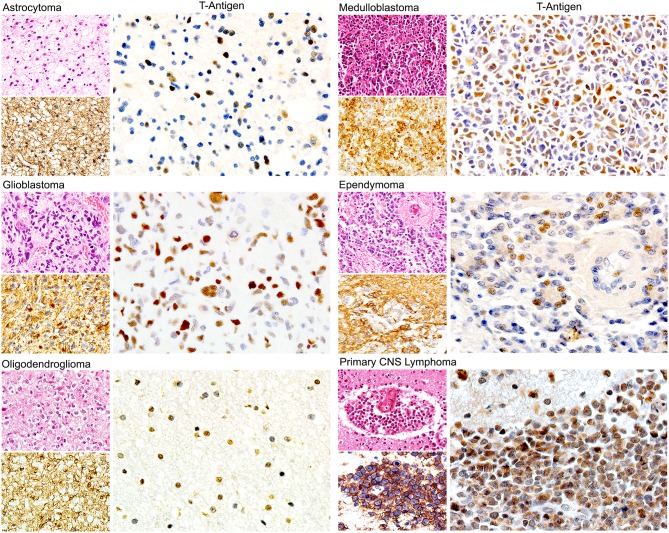
Expression of JCPyV T-Antigen in human brain tumors. Immunohistochemical studies have demonstrated the expression of the viral oncoprotein in a wide variety of astrocytoma, oligodendroglioma, ependymoma to Glioblastoma. Other tumors derived from the neural crest, such as medulloblastomas are also positive. Interestingly, primary CNS lymphomas are also positive. Expression of T-Antigen is nuclear, and interestingly not all neoplastic cells are positive. An example of each of these tumors is shown, with their Hematoxylin and Eosin and specific marker, which for glial tumors is GFAP, for medulloblastomas is synaptophysin and for CNS lymphomas is CD20. (All panels original magnification is 600x).

Since, in addition to glial cells, JCPyV has been shown to infect B-lymphocytes, some studies have investigated the presence of the Polyomavirus in primary CNS lymphomas, which are also tumors seen with high frequency on patients with AIDS. In the first of these studies, viral genomic sequences of all JCPyV regions were amplified in 81% of the 27 cases studied, although expression of T-Antigen was detected in only 18.5% (149). Interestingly, JCPyV T-Antigen was always co-localized with the EBV latent protein LMP1, suggesting a cross-talk between the two viruses; however, this notion needs to be further explored. In addition, several reports have shown the concomitant PML and primary CNS lymphomas (156–158), suggesting that, like in PML, the presence of HIV-1 is important in the activation of JCPyV. Figure 4 depicts the expression of nuclear T-Antigen in neoplastic cells of different phenotypes of brain tumors.

### The Merkel and Raccoon Polyomaviruses

During the last decade, two novel and groundbreaking discoveries have helped cement the role of Polyomaviruses in oncogenesis. The first one was the discovery of a new Polyomavirus in Merkel cell carcinomas (159), which, although in a different branch of the *Polyomaviridiae* family, seems to use similar mechanisms as JCPyV in its malignant transformation of cells, such as the T-Antigen “hit and run” mechanism. The second was the discovery of a new racoon Polyomavirus (RacPyV1), which was isolated from brain tumors of racoons in northern California (160), constituting the first time that a Polyomavirus has been linked to a tumor in an animal on its natural state. The 10 raccoons were non-family members, clustered in the same area. The tumors developed were all malignant and glial in origin and were all located in the frontal lobe in close proximity to the olfactory nerve, which suggested a respiratory via on entry for the Polyomavirus. RacPyV1 was isolated from all 10 tumors, but it was found to be episomal, rather than integrated. No viral amplification was found in other tissues, neither in 20 additional raccoons from the same area who died of different causes. Later, it was demonstrated by the same group that the oncogenicity of RacPyV1 is also due to the production of T-Antigen, although this occurs via alternative splicing and the transcription was significantly larger in the tumors than other cellular host genes. Furthermore, glioblastomas are not naturally occurring tumors in raccoons, strongly suggesting RacPyV as an oncogenic virus and the etiological agent of these neoplasms.

### JCPyV in Colon Cancer and Other Human Tumors

While the role of JCPyV in the pathogenesis of brain tumors has been intensely debated, its role in the pathogenesis of colon cancer has been extensively documented and is less controversial. As discussed above, JCPyV genomic sequences have been found in epithelial cells of the gastrointestinal tract (161). This study was pivotal for two reasons, first, it showed that treatment with topoisomerase improved the yield of viral genome detection, suggesting that the DNA is negatively supercoiled, which may be another reason of the lack of success detecting JCPyV in early studies on brain tumors; and second, it opened the door for an ample body of work demonstrating the presence of JCPyV in colonic pre-neoplastic lesions and malignancies (162–164). As in brain tumors, the study by Enam demonstrated that the JCPyV genomic sequences were amplified from tumor cells micro-dissected from the tissue using laser capture. Such studies have been successfully reproduced in different ethnic groups of people and in countries al around the world, including Japan (165), China (166, 167), Taiwan (168), Israel (169), Portugal (170), and Tunisia (171). The multiple studies on colon cancer have also been important for several reasons; they have shown that preneoplastic polyps and adenocarcinomas contain up to 20 × 10^3^ viral copies per microgram of DNA, while adjacent colonic mucosa and normal colon have only a low viral load (172), implicating JCPyV in the process of neoplastic transformation. They have also shown different molecular and cellular pathways affected by JCPyV, providing strong evidence of oncogenic mechanisms utilized by the virus. In one of these studies it was shown that T-Antigen was detected in 92% of primary tumors and 73% of liver metastases, and that expression of the oncogenic protein *in vitro* resulted in the activation of cellular genes that mediate cell motility and invasion. Specifically, inhibition of the PI3K/AKT and MAP Kinase pathways that were activated by T-Antigen, resulted in significantly reduced migration and invasion (173). Another pathway affected by JCPyV is the Wnt signaling pathway; several studies have shown that T-Antigen can bind to β-catenin and translocate it to the nucleus, where it activates the transcription of cell cycle regulators c-Myc and Cyclin D1, causing cells to proliferate (174).

But perhaps the most significant contribution of these mechanistic studies is the corroboration that genomic instability is caused by JCPyV infection. It was shown that colonic epithelial cells exposed to the Mad 1 and Delta98 strains of JCPyV developed chromosomal breakages, duplications, dicentric chromosomes and increased polyploidy, at time point of 7, 14, and 21 days after transfection (175). Cells do not survive further as this is the time frame of the lytic cycle of the virus. In addition, a large study in which expression of T-Antigen was found in 43% of 100 colorectal carcinomas, demonstrated a significant association between the oncogenic protein and microsatellite instability and the *de novo* methylation of the promoter regions of nine putative tumor suppressor genes associated with colon cancer (176). An even larger study in 766 cases of colorectal carcinoma corroborated these findings and showed that T-Antigen, expressed in 35% of the tumors correlated with DNA methylation of 8 critical promoters of transcription and proliferation factors (CACNA1G, CDKN2A, CRABP1, IGF2, MLH1, NEUROG1, RUNX3, and SOCS1), and with microsatellite instability (177).

**Figure 5:**
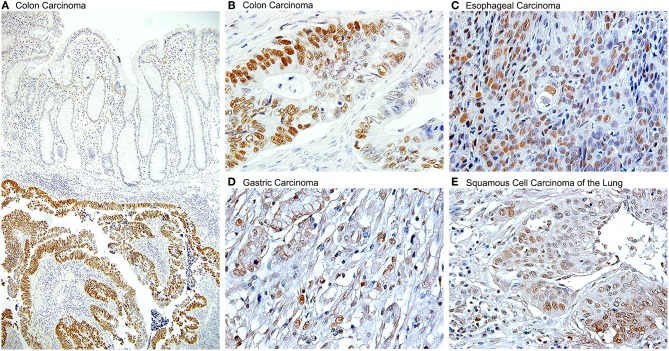
Detection of JCPyV T-Antigen in various human tumors. Immunohistochemistry for the JCPyV early product, large T-Antigen shows robust expression in a variety of epithelial human cancers. **(A)** At low magnification, the expression of T-Antigen is evident in the submucosal tumor, however the suprajacent normal colonic epithelium is completely negative (Original magnification 100x). **(B)** At higher magnification, the majority, but not all neoplastic cells shown nuclear T-Antigen (Original magnification 600x). Other tumors in which T-Antigen is present are **(C)** esophageal carcinomas, **(D)** gastric carcinomas, and **(E)** non-small cell carcinomas of the lung, including both, adenocarcinomas and squamous cell carcinomas, as the one shown (Original magnification for the last three panels 400x).

The evidence of the role of JCPyV in the pathogenesis of a large number of colon cancer cases has been convincing enough that a new line of thinking is leading to use JCPyV as a tool for determining high risk and early detection of colon cancer. It has been suggested that serum antibodies against viral proteins can be used as a factor for high risk in developing colorectal polyps or sporadic cancers (178); and detection of JCPyV specific miRJ1-5p in feces has been proposed as a novel biomarker for non-invasive screening of colon cancer (179). The ability of JVPyV to infect epithelial cells from the gastrointestinal tract in a non-productive manner, and the strong body of evidence showing its association with pre-neoplastic polyps and colorectal carcinomas opened the door for investigating the presence of JCPyV genomic sequences and expression of T-Antigen in a variety of other gastrointestinal tumors, which have now been reported in gastric carcinomas (180–182), esophageal carcinomas, where it utilizes the same mechanisms as in brain tumors and colon cancer, which is the binding and inactivation of p53 and the disruption of the Wnt signaling pathway (150), and as a risk factor for carcinomas of the tongue (183). Since one of the sites of JCPyV latency is the kidney, recent studies are also demonstrated the presence of the Polyomavirus in bladder carcinomas (184, 185), where the control region of the archetype (CY) strain and other rearranged strains have been amplified. And finally, association of JCPyV also been reported in other malignant epithelial tumors, such as non-small cell carcinomas of the lung (186). Figure 5 shows the detection of JCPyV in different types of epithelial tumors, including colon carcinomas.

### T-Antigen and Mechanistic Studies

While the majority of the studies reviewed above have shown the presence and association of JCPyV with brain tumors, colon cancer and other human malignancies, as it has been said many times, casualty does not prove causality, therefore, mechanistic studies were needed in order to prove the role of Polyomaviruses in oncogenesis. Since JCPyV is a small virus with a limited number of genes, the candidates to mediate the transforming abilities of the virus are limited. The late coding region encodes for 4 proteins, 3 of which are structural capsid proteins, and one small accessory protein, Agno. On the other side, the early transcriptional region encodes for 1 protein, large T-Antigen, the main regulatory protein, and 4 spliced versions. In addition to regulate the JCPyV life cycle and prepare the machinery of infected cells for viral replication and capsid production, T-Antigen interacts and dysregulates several cellular processes that, under specific circumstances, such as an abortive, non-lytic infection, immune evasion, and integration, leads to the malignant transformation of non-permissive cells.

T-Antigen is a relatively small, but versatile nuclear phospho-protein of 688 amino-acids (187), that is long known to have binding sites for the key cell cycle regulators p53 (34, 35), and pRb (188, 189), and by sequestering them and inactivating them, induces cells to proliferate. In the case of Rb, it is believed that the interaction with T-Antigen also activates E2F transcription factors that promote cell cycle progression (190). Another pathway targeted by JCPyV is the Wnt signaling pathway. The central domain of T-Antigen located between amino-acids 82–628 can bind to the C-terminus of β-catenin (191), a protein normally located and degraded in the cytoplasm, resulting in its stabilization and eventual nuclear translocation, where it enhances TCF promoter activity and activation of c-Myc and Cyclin D1, driving cellular proliferation (174). Adjuvant to this process, T-Antigen can also recruit the GTPase protein Rac1 to stabilize β-catenin by inhibiting its ubiquitin-dependent proteasomal degradation, and allowing for its nuclear translocation (192).

As it was discussed in colon cancer, JCPyV infection has been shown to cause direct DNA damage and genomic instability. The first evidence of this chromosomal damage was discovered in lymphocytes in was denominated the “rogue cell” theory (193, 194), in which numerous chromosomal aberrations in lymphocytes are present in individuals exhibiting high antibody titers for JCPyV. But it has also been demonstrated that infection with JCPyV in glial cultures can induce numerous insults to DNA, including polyploidy, and especially double stranded breaks (DSBs), directly related to the duration of the infection (195, 196). Damaged cells can repair DSBs by two mechanisms, homologous recombination (197), a faithful process which requires significant energy, cell division, since it requires an homologous DNA template, and involves the enzyme Rad51; however, if this process cannot take place, damaged cells are forced into non-homologous end-joining, a much economic metabolic process in which loose ends of DNA are simply attached (198). This process, which does not require significant energy expenditure, cell division or a homologous template, utilizes the Ku70/Ku80 complex, and usually results in the accumulation of mutations. A crucial and fortuitous discovery was the binding of JCPyV T-Antigen to another cellular protein, IRS-1 (Insulin Receptor Substrate 1), the downstream docking molecule if the IGF-1R pathway. As it happened with β-catenin, this process resulted in the nuclear translocation of IRS-1 (199). The presence of IRS-1 in the nucleus, which had never been reported at the time, had a surprising effect in cells that were undergoing DNA damage by the presence of JCPyV. IRS-1 binds and inactivates the above-mentioned enzyme Rad-51, preventing faithful homologous recombination and forcing these cells to repair their double stranded breaks via non-homologous end-joining (200). In addition, it appears that ectopic expression of the JCPyV accessory protein Agno also affects the response of cells to DNA damage. Cells expressing agnoprotein were more sensitive to the cytotoxic effects of cisplatin and exhibited increased chromosome fragmentation, micronuclei formation, and impaired DNA damaged-induced cell cycle arrest (196). Finally, a very recent article shows that JCPyV T-Antigen also recruits host DNA damage response proteins to nuclear sites or viral replication, including PATM, a kinase that signals MRN complex to double stranded breaks, Rad 50, another repair enzyme with similar functions to Rad51, RPA80, a replication protein complex, and TopoI/TopoIIβ, proteins that help promote the supercoiling of repaired DNA, further altering faithful DNA repair (201).

Apoptosis is a homeostatic mechanism to dispose of senescent or damaged cells, including virally infected cells. In the damaged group, cells that cannot repair the double stranded breaks via homologous recombination or non-homologous end-joining, also activate their programmed death mechanism. Interestingly, despite their extensive DNA damage, caused by JCPyV infection and their alterations in DNA repair mechanisms, there is no evidence of apoptosis in infected cells in cases of PML. This may be explained by the discovery of another pathway targeted by JCPyV in order to complete its replication cycle. It has been demonstrated that T-Antigen is capable to activate the Survivin promoter. Survivin is a potent anti-apoptotic protein, normally expressed during embryonic development, but completely silenced in fully differentiated tissues (202). Infection of glial cell cultures with JCPyV resulted in a significant production of Survivin, which in turn protected infected cells for undergoing spontaneous and induced apoptosis (121). This T-Antigen mediated process may also play an important role in oncogenesis. Since, as discussed above, T-Antigen cooperates with IGF-1 receptor in the transformation of neuronal progenitors in the cerebellum, leading to the formation of medulloblastomas, we explored the possibility of Survivin being involved in this process. IGF-IR knockout embryos showed decreased levels of Survivin in the brain, compared to their non-transgenic littermates, resulting in elevated apoptotic neuronal precursors and a poorly differentiated phenotype. In addition, in wild type IGF-IR neural progenitors *in vitro* induction of T-Antigen expression tripled the expression of Survivin and accelerated cell proliferation, in contrast with knockout progenitors, in which induction of T-Antigen failed to increase Survivin, resulting in massive apoptosis (203), suggesting that reactivation of the normally dormant Survivin by T-Antigen may contribute to the transformation process induced by JCPyV infection.

The main role of small t-Antigen in transformation is to bind and inactivate the protein phosphatase 2A (PP2A), a serine/threonine phosphatase that regulates phosphorylation signals activated by kinases, and has been shown to function as a tumor suppressor gene in a variety of cancers (204). JCPyV small t-Antigen, through its interaction with PP2A, interferes with nucleotide excision repair, contributing to the alterations on DNA repair mechanisms (205). The region of small t-Antigen that has binding sites for PP2A is not present in large T-Antigen, however, the end result of this binding is the same as the one exerted by its larger version. Inactivation of PP2A results in the inhibition of the Wnt signaling pathway and nuclear translocation of β-catenin, perhaps carried by large T-Antigen as discussed above, stimulating cell proliferation genes. In addition, inactivation of PPA2 by small t-Antigen seems to induce alterations in cytoskeletal proteins, mainly actin, and in tight junctions, resulting in the loss of cell polarity and in increased invasiveness (206). Finally, as for the contribution of the splice variants, the function of T'135, T'136, and T'165 is not completely understood, however, they have been shown to bind to the Retinoblastoma pocket proteins p107 and p130, altering their phosphorylation status, and promoting cell cycle progression through activation of E2F transcription factor activity (38).

## Discussion

Since the Nobel award discovery of the retrovirus that causes sarcomas when transmitted into healthy chicken (207), the association of viruses with cancer has been well-established. It is estimated that 12–20% of human cancers are caused by viral infections. In particular some DNA viruses are considered oncogenic; Epstein Barr virus (EBV) is the cause Burkitt's lymphoma, certain types of Hodgkin lymphoma and nasopharyngeal carcinoma (208), Human Herpesvirus Type 8 is the cause of Kaposi's sarcoma and primary effusion lymphoma (209–211), and human papilloma viruses are known to cause nearly all cases of cervical cancer (212). But, despite the well-characterized transforming abilities of Polyomaviruses *in vitro*, and the established oncogenic potential of JCPyV in animal models, either after direct inoculation of JCPyV or expression of the transgene encompassing the JCPyV early genome in transgenic animals, the association of JCPyV with human tumors has been intensely debated during the last decade. While several studies have successfully detected viral genomic sequences by PCR and viral proteins by immunohistochemistry in several types of brain tumors, lymphoid tissue, and other organs including the gastrointestinal, urinary and respiratory tracts, some other minority of projects have failed to establish the presence of JCPyV in the same types of human tumors (213). Two more recent studies in which deep sequencing was used to detect a wide variety of viral transcripts, failed to detect JCPyV activity in low grade gliomas (57 and 100, respectively), glioblastomas (168 and 162, respectively), and colon carcinomas (138 and 407, respectively). The first study used deep sequencing of RNA (RNA-Seq) (214), while the second study was a computational analysis of whole genome, exome, and RNA-Seq from existing databases provided by the Cancer Genome Atlas (215). These discrepancies may be due to technical difficulties and especially to the quality of the available tissue. For example, it is well known that long periods of fixation in formalin contribute to DNA degradation making PCR amplification challenging (216, 217). Other methodological issues including, the length of time that tissue has been stored, the efficiency of DNA extraction, the sensitivity of PCR and Southern blot, and the copy number of positive controls used in the assays may also contribute to the observed discrepancies in the detection of viral DNA in paraffin-embedded archival samples. These issues may be of particular importance for archival databases, like the one used in the second study discussed above.

**Figure 6:**
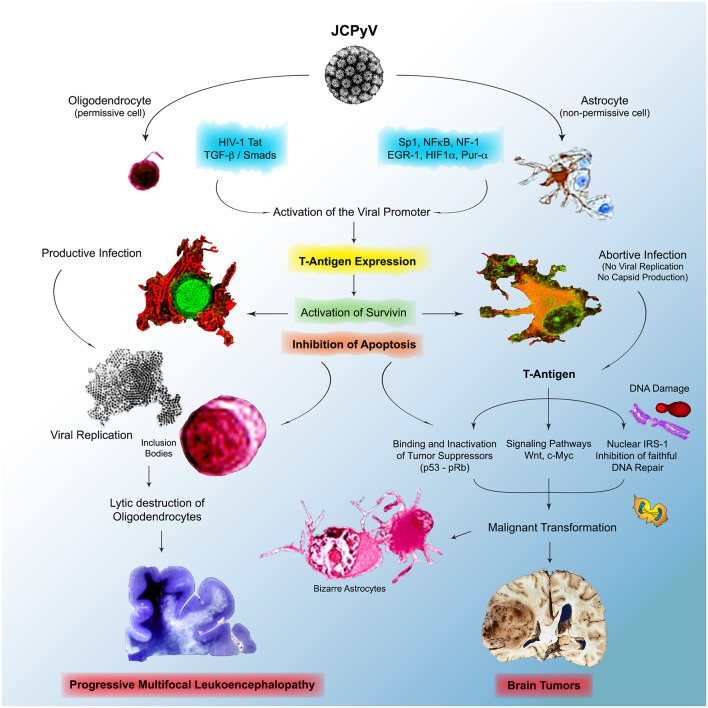
Physiopathology of JCPyV in the brain. JCPyV enter the brain and infects both phenotypes of glial cells, oligodendrocytes and astrocytes, which results in the activation of the normally dormant protein Survivin, which results in the inhibition of apoptosis, a mechanism to dispose of virally infected cells. In oligodendrocytes, permissive to active infection, this allows time for JCPyV to complete its life cycle, which results in active viral replication in the nuclei, the formation of inclusion bodies, and the lytic destruction of the myelin producing cells end in Progressive Multifocal Leukoencephalopathy. In astrocytes, where the infection is abortive, no viral replication takes, but the viral oncoprotein T-Antigen is expressed. T-Antigen binds and inactivates p53 and pRb, crucial tumor suppressors and can affect important cell cycle regulator pathways, including the Wnt signaling pathway, resulting in the activation of c-Myc. Infection with JCPyV also causes DNA damage, and by binding to IRS-1, the downstream protein if the IGF-1 pathway, it inhibits faithful DNA repair, forcing cells to repair their DNA via non-homologous end-joining, which results in mutations. All these are contributing factors of cellular transformation, which in certain individuals may results in the development of brain tumors.

However, the multitude of mechanistic studies during the last 2 decades has shed much needed light into the pathogenesis of JCPyV, yet multiple events are still not fully understood. We do know that JCPyV is a ubiquitous virus that infects the majority of the human population world-wide, and that the archetype strain of the virus remains in a latent state in several cells and organs, including lymphocytes, the bone marrow, and epithelial cells in the kidney. We also know that lymphocytes, in which JCPyV causes chromosomal abnormalities and breaks are carriers of the virus into the brain; these cells are also likely the place where mutation and rearrangements in the control region occur to give origin to strains associated with PML and found in brain tumors. Although JCPyV is episomal in cases of PML, allowing for the presence of all genomic regions of the virus and for the production of viral capsids during the late phase of infection, is not clear where the presence of viral DNA is episomal or integrated in brain tumors, although evidence suggest integration into the cellular DNA, and in these cases, T-Antigen can be expressed without capsid production or viral particle formation. We also know that the JCPyV infects humans exclusively, with a limited cell and tissue tropism, and that gene expression and DNA replication are restricted by factors in the regulatory region that are species- and tissue specific (218). In the human adult brain JCPyV infects glial cells with very different outcomes. Infection of astrocytes, non-permissive cells is abortive, while infection of oligodendrocytes, permissive cells, is productive and results in the lytic destruction of the myelin producing cells in the CNS. With mechanistic studies we envision a scenario in which, after immune evasion and infection, the initial expression of T-antigen results in the activation of the normally dormant Survivin promoter, which inhibits apoptosis in both types of cells. In oligodendrocytes, this allows time for JCPyV to complete is life cycle, with DNA replication, late gene activation with production of viral capsids, viral assembly in the nucleus, which is the basis for the eosinophilic viral inclusion bodies, and the eventual lysis of oligodendrocytes, leading to demyelination plaques and PML. On the other hand, in astrocytes, the activation and production of Survivin, prevention of apoptosis and increased life span, maybe allow for T-Antigen time to bind and inactivate cell cycle regulators like p53 and pRb, and for the host cell DNA to undergo damage, including polyploidy, double stranded breaks. It would also allow time for T-Antigen to bind and translocate IRS-1 to the nucleus, where it binds to Rad51, impeding faithful homologous recombination DNA repair, pushing cells to repair their damaged DNA via non-homologous end-joining, resulting in the accumulation of mutations. In the case of PML, the result of these processes may be the bizarre, multinucleated astrocytes, however, because the very short life span of patients with PML, the process ends there, but in other conditions, that may be a contributing factor in the malignant transformation of astrocytes that may lead to the formation of glial tumors. A similar scenario may be also at play in neuronal progenitors, resulting in the formation of medulloblastomas, and in epithelial cells of the gastrointestinal tract, where productive infection does not occur; the prevention of apoptosis may result in time for T-Antigen to dysregulate the Wnt signaling pathway, by binding and translocation β-catenin to the nucleus, where it activates c-Myc and Cyclin D. Figure 6 depicts a diagram of this hypothetical model of mechanisms utilized by JCPyV in both, PML and brain tumorigenesis.

Overall, the mounting available data and mechanistic studies suggest that JCPyV, under immunosuppressive conditions causes PML, however under circumstances that we don't fully understand, including the role of the immune system, may give origin to clones of cells that are driven to malignant transformation. It would be naïve and arrogant to consider JCPyV as the cause of brain tumors, colon cancer or other malignancies, since for instance, there are a good percentage of tumors in which neither JCPyV and T-Antigen are detected. However, it is time to give JCPyV its rightful place as a contributing factor in the pathogenesis of human tumors.

## Author Contributions

LD wrote the majority of the manuscript, prepared half of the figures, table, and all references. SP-O wrote portions of the manuscript and prepared half of the figures.

### Conflict of Interest Statement

The authors declare that the research was conducted in the absence of any commercial or financial relationships that could be construed as a potential conflict of interest.
